# Anammox-MBR Technology: Breakthroughs and Challenges in Sustainable Nitrogen Removal from Wastewater

**DOI:** 10.3390/membranes15110337

**Published:** 2025-11-10

**Authors:** Sumayya Abdul Rahiman, Hazim Qiblawey

**Affiliations:** Department of Chemical Engineering, Qatar University, Doha P.O. Box 2713, Qatar; sa2314916@student.qu.edu.qa

**Keywords:** anammox, membrane bioreactor, nitrogen removal, wastewater treatment, membrane fouling, microbial ecology, partial nitrification, sustainable technology

## Abstract

Wastewater nitrogen pollution is a serious environmental problem, and traditional treatment techniques are frequently constrained by their high energy requirements and operational complexity. The anaerobic ammonium oxidation (anammox) process combined with membrane bioreactor (MBR) technology (anammox-MBR) offers a practical and energy-efficient solution for the sustainable removal of nitrogen, further enhanced by its potential to minimize emissions of nitrous oxide (N_2_O), a potent greenhouse gas with a global warming potential nearly 300 times that of carbon dioxide. This review outlines the most recent advancements in anammox-MBR systems, highlighting their ability to achieve nitrogen removal efficiencies of more than 70–90% and, in integrated systems with reverse osmosis, to recover up to 75% of the inflow as high-quality reusable water. Significant advancements such as high-rate activated sludge coupling, reverse osmosis integration, microaeration methods, and membrane surface modifications have decreased membrane fouling, accelerated startup times, and enhanced system stability. Despite these achievements, there are still issues that hinder widespread use, such as membrane fouling exacerbated by hydrophobic anammox metabolites, sensitivity to low temperatures (≤10 °C), and the persistent challenge of suppressing nitrite-oxidizing bacteria (NOB), which compete for the essential nitrite substrate. To enable cost-effective, energy-efficient, and environmentally sustainable large-scale applications, future research directions will focus on creating cold-tolerant anammox strains, advanced anti-fouling membranes, and AI-driven process optimization.

## 1. Introduction

Nitrogen-rich wastewater is a major environmental issue that requires proper treatment before disposal. The traditional nitrification-denitrification process requires substantial energy inputs and generates more sludge than the anammox process [1]. The discovery of anaerobic ammonium-oxidizing (anammox) bacteria introduced a highly efficient, low-sludge nitrogen removal technology. Due to their slow growth, anammox bacteria require systems that maintain a very high solids retention time (SRT), which can be achieved using technologies like membrane bioreactors (normally 12 days and even 30–1280 days) compared to conventional methods [2,3,4]. The combination of anammox with membrane bioreactor (MBR) technology provides a controlled operational environment that enables faster growth rates and high-purity enrichment of anammox cultures. This is demonstrated in Membrane-Aerated Biofilm Reactors (MABRs), where a erasequential aeration strategy was shown to selectively enrich for anammox bacteria while suppressing nitrite-oxidizing bacteria, leading to high nitrogen removal rates of up to 5.5 g N m^−2^ d^−1^ (at loads up to 8 g N m^−2^ d^−1^) [5]. The absence of settling pressure, along with low shear stress and balanced nutrient conditions, supports the development of completely suspended anammox cells [6]. Additionally, MBR systems facilitate the selective enrichment of specific anammox strains based on substrate affinity [4]. The key benefits of this integration include the ability to achieve high nitrogen removal efficiencies of over 70–90% and recover a significant portion of wastewater as high-quality effluent. However, critical challenges such as membrane fouling, temperature sensitivity, and microbial competition hinder widespread application, necessitating this review to combine recent solutions and identify existing research gaps.

Among these, managing microbial competition is paramount. The successful implementation of anammox, particularly in mainstream wastewater treatment, is critically dependent on suppressing nitrite-oxidizing bacteria (NOB). NOB outcompete anammox bacteria for nitrite (NO_2_^−^), converting it to nitrate (NO_3_^−^) and thereby undermining the stoichiometric balance required for efficient anaerobic ammonium oxidation. Developing reliable strategies for NOB suppression remains a significant research frontier [7].

To ensure a comprehensive and representative analysis, the literature was systematically sourced from primary databases (Scopus, Web of Science) using targeted queries for anammox-MBR configurations, performance, and challenges, with a focus on peer-reviewed research and review articles from the past years. This approach offers a clear framework for evaluating the progress and representativeness of the findings covered in this article.

Membrane-aerated biofilm reactors (MABRs) integrated with anammox processes have further improved nitrogen removal efficiencies, achieving over 70% removal with low NO and N_2_O emissions (<1.0% of total nitrogen (TN) removed) by optimizing oxygen supply, ammonium loading rates, and biofilm thickness. Compared to conventional biofilm systems, MABRs produce significantly less N_2_O, positioning them as a promising and sustainable technology for advanced nitrogen removal in wastewater treatment [6]. Recent advances highlight simultaneous nitrification, anammox, and denitrification (SNAD), partial denitrification/anammox (PD/A), and nitrate denitrifying anaerobic methane oxidation-anaerobic ammonia oxidation (DAMO-anammox) as the most viable configurations for mainstream implementation, addressing key bottlenecks like low-temperature operation (<20 °C), nitrite-oxidizing bacteria (NOB) suppression, and nitrate accumulation. Emerging systems now enable resource recovery while treating low C/N wastewater, though full-scale adoption requires integrated engineering solutions. For example, combining acid-tolerant bacteria with an MABR has proven to be a robust solution for achieving the stable process control necessary for nitrogen removal, a key step in these systems [8]. A novel acidic MABR achieved breakthrough partial nitritation for mainstream wastewater, creating ideal conditions for a subsequent anammox process. By operating at pH 5.0–5.2 with acid-tolerant bacteria *Candidatus Nitrosoglobus*, it suppressed nitrite-oxidizers for >200 days while achieving record-high ammonia removal (2.4 kg N m^−3^ d^−1^) at just 30 min retention time [9].

**Figure 1:**
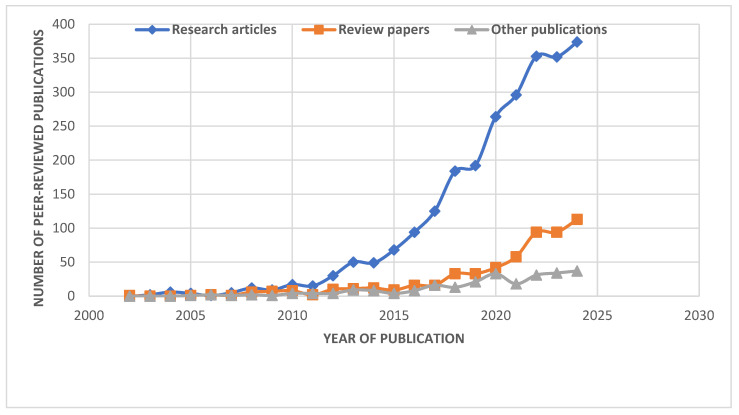
Evolution of Anammox-MBR Research (2000-Present). The data was obtained from Scopus using the systematic search strategy detailed in Section 1, reflecting the growing research interest in this technology. The *y*-axis represents the annual number of publications (including research articles and reviews using the query—All fields (“anammox MBR”) (search date: 28 August 2025; document types: Article, Review, Others)).

This review reveals how newly discovered bacteria treat tough wastewaters (salty, acidic, contaminated) [1,9], proves real-world success and cost savings in large plants [10], and introduces cutting-edge solutions like AI fouling control and carbon-neutral designs [11,12]- filling gaps left by earlier reviews. Special attention is given to breakthroughs such as rapid partial nitrification/anammox (PN/A) initiation through microaerobic cultivation, NOB suppression strategies, and innovative membrane modifications, including visible-light responsive photocatalytic membranes for in situ biofouling mitigation [13,14]. The figure below (Figure 1) shows the evolution of Anammox-MBR research in recent years. Steady growth since 2015 indicates rising global interest. Recent trends show an increasing focus on research articles.

## 2. Anammox Process—Biochemistry

**Figure 2:**
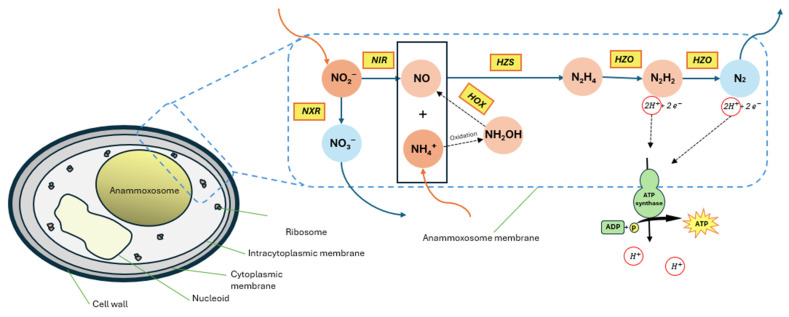
Schematic representation of anammox mechanism—Redrawn, inspired by the works of [23,24].

Anammox bacteria use nitrite (NO_2_^−^) as the electron acceptor to convert ammonium (NH_4_^+^) straight to nitrogen gas in anaerobic conditions [15,16]. Compared to conventional nitrification-denitrification systems, Anammox-based MBRs are more energy-efficient because the process is autotrophic, requires no external organic carbon source, and operates under anaerobic or low-oxygen conditions, significantly reducing aeration energy demand [17]. In MBRs containing limited non-Anammox populations and free Anammox bacteria, the stoichiometry has been confirmed [17]. It offers superior nitrogen removal while eliminating the need for external organic carbon and significantly reducing the energy-intensive aeration required by conventional processes, making it especially useful for effluents with high ammonia and low C/N ratios [18]. Using hydrazine (N_2_H_4_) as a central catabolic intermediate in the oxidation of ammonium, which is produced inside the anammoxosome, a specialized organelle only found in Anammox bacteria, is one of its distinctive features [19]. The anammox cell is compartmentalized by three lipid membrane layers, which enhance reaction efficiency by maintaining proton gradients essential for energy conservation. Anammox metabolism includes a series of reactions catalyzed by different enzymes (Figure 2).

The overall anammox reaction or the energy-generating process in an anammox cell is given in Equation (1) [15].

(1) $${{NH}_{4}^{+}+1.32{NO}_{2}^{-}+0.066{HCO}_{3}^{-}+0.13H^{+}\to 1.02N_{2}+0.26{NO}_{3}^{-}+0.066{CH}_{2}O}_{0.5}N_{0.15}+2.03H_{2}O$$

Enrichment is essential since anammox bacteria grow slowly (doubling time: 7–22 days) [20]. Optimized reactor conditions, technologies that facilitate biomass retention, and bioaugmentation with pre-enriched sludge are some ways to speed the start-up [21]. High cell density and resilience have been shown by granular sludge and biofilm carriers, increasing reactor performance and stability [22].

## 3. Microbial Ecology and Community Dynamics

Microbial ecology significantly affects nitrogen removal and stability in anammox-MBR systems. A novel selection technique, leveraging the MBR’s long sludge retention time (SRT) and high mixed liquor suspended solids (MLSS) to selectively enrich highly active anammox consortia, quadrupled the anammox growth rate (0.334 d^−1^) and tripled ammonium uptake without changing *Candidatus Brocadia* dominance. This demonstrates that growth kinetics are highly influenced by these operational conditions [25]. Oxygen, substrates, and reactor design all affect microbial populations, which in turn affect aggregation and succession. Highly active anammox cultures are enriched by controlled cultivation in systems like membrane bioreactors (MBRs). This method creates a free-cell suspension that eliminates mass transfer limitations, enabling the expression of intrinsic high growth rates (up to 0.21 d^−1^) and revealing an extremely high affinity for nitrite (half-saturation constant, Ks, of 0.035 mg N L^−1^), thereby optimizing nitrogen removal [17].

Functional compartmentalization allows microbial consortia to adapt to stressors like bisphenol A (BPA), with heterotrophs inhabiting flocculent sludge and *Candidatus Kuenenia* dominating membrane sludge, which upregulates denitrification genes to mitigate enzymatic inhibition [26]. Maintaining balanced ratios of ammonia-oxidizing bacteria (AOB) and anammox bacteria, while suppressing NOB, is vital [27]. While heterotrophs minimally affect nitrogen removal, the anammox bacteria themselves are a major cause of membrane fouling. Their metabolites contain abundant hydrophobic groups (e.g., CH_3_, CH_2_, CH), which readily deposit on the membrane surface. Embedding magnetic porous carbon microspheres as carriers mitigates this fouling by adsorbing these hydrophobic compounds and immobilizing the bacteria, thereby extending membrane life [28]. Suspended carriers promote nitrogen removal, decrease membrane fouling, and increase system stability by promoting the formation of dense biofilms or granules, decreasing microbial byproducts, and providing continuous mechanical scouring that mitigates membrane fouling without causing significant biomass washout [29]. Carbon-based carriers stabilize biofilms at low temperatures and organic loads by improving electron transfer and adsorbing inhibitors for mainstream applications [29].

The availability of substrates affects both microbial activity and stability. Low nitrogen promotes biomass growth by increasing macromolecule synthesis and enabling energy-efficient nitrogen removal [30]. *Candidatus Brocadia sapporoensis* thrives in propionate-rich wastewater using mixotrophy and dissimilatory nitrate reduction to ammonium (DNRA), and it dominates low C/N conditions through efficient propionate metabolism [31]. Anammox bacteria show metabolic versatility, using organic compounds and nitrate-to-ammonium reduction under stress to maintain resilience and nitrogen removal [32]. Quorum sensing may regulate biofilm formation and extracellular polymeric substances (EPS) secretion, offering a potential strategy to enhance resilience under inhibition [33].

Reactor design, especially MABRs, affects partial nitritation efficiency. In counter-diffusion biofilms (e.g., in an MABR), oxygen is supplied from the bottom through a membrane, while substrates (NH_4_^+^) diffuse from the top. In contrast, in co-diffusion biofilms (conventional biofilms), both oxygen and substrates diffuse from the bulk liquid at the top. Counter-diffusion biofilms outperform co-diffusion biofilms, with AOB and NOB growth rates primarily determining performance [34]. Effective NOB suppression strategies exploit physiological differences: low DO (<0.5 mg L^−1^) inhibits NOB more than AOB [7], and chemical inhibition via free ammonia selectively targets NOB [35].

Pilot-scale integration of activated sludge, reverse osmosis (RO), and PN/A achieved 75% water recovery from the RO unit, producing high-purity permeate. The remaining 25% of the inflow was concentrated as a reverse osmosis concentrate (ROC) stream, which was subsequently treated for nutrient removal via the PN/A process. The system demonstrated >85% nitrogen removal, and 95% phosphorus recovery without the use of chemicals, resulting in a 26% reduction in energy consumption and enabling sustainable reuse [36]. Efficient PN to PN/A transitions with microaerobic pre-cultivated sludge ensure stable, high-rate nitrogen removal for practical mainstream applications [13]. Suspended carriers enhance nitrogen removal, reduce fouling, and stabilize anammox-MBRs by promoting biofilms, lowering soluble microbial products and EPS, and providing mechanical cleaning to improve system lifespan and efficiency.

## 4. Process Configurations, Performance, and Membrane Fouling Control

### 4.1. Process Configurations and Performance

A critical comparison of these configurations reveals both agreements and contradictions in the literature. There is strong agreement on the robustness of PD/A systems, with multiple studies confirming their high nitrogen removal (93–98%) across a wide temperature range (10–40 °C) and their suitability for low C/N wastewaters [37]. In contrast, reports on PN/A stability are more mixed. While some studies demonstrate rapid initiation within 19 days and stable operation by controlling DO and pH [35,38], others highlight its persistent sensitivity to NOB competition and temperature, presenting it as a key limitation [7,37]. This discrepancy underscores that PN/A success is highly dependent on precise, real-time operational control. Furthermore, a clear trade-off is evident between simpler suspended growth systems [6] and more complex biofilm carriers [30,39]. While carriers enhance stability and reduce fouling, they add complexity and cost, indicating that the optimal configuration is wastewater specific.

Recent studies have demonstrated that partial nitritation (PN) can initiate in MBR systems within 19 days by maintaining dissolved oxygen (DO) levels between 0.8 and 0.9 mg L^−1^. This results in nitrite-rich water with a favorable nitrite-to-ammonia ratio (~1.15) for anammox activity. While *Nitrosospira* sp. and *Nitrosomonas* predominate as AOB during various phases, providing stability, free ammonia suppresses NOB. However, elevated levels of ammonium cause a rise in EPS, which promotes membrane fouling. Therefore, maintaining operational stability and minimizing fouling requires regulating DO, pH, and nitrogen levels [35,40].

The reverse anammox-partial nitrification system using a fixed-bed reactor and MBR removed over 90% nitrogen and chemical oxygen demand (COD). It worked well with low carbon-to-nitrogen (C/N) ratios and high ammonium loads, with nitrogen removal rates between 0.92 and 1.03 kg N m^−3^ d^−1^. Performance improved by optimizing the internal recycling ratio [40]. Carbon-based biocarriers like biochar and activated carbon helped stabilize the system by adsorbing inhibitory compounds, supporting slow-growing microbes, and enabling direct electron transfer [7].

Several hybrid systems have performed well. The SNAD process in oxygen-limited MBRs with biogas recirculation removed 94.86% total nitrogen and 98.91% COD, while reducing the membrane fouling rate [41]. Methane-based systems achieved 60% nitrogen removal and 95% methane use, allowing energy recovery during treatment [42].

A moving bed biofilm reactor (MBBR) used for municipal wastewater treatment achieved 96.7% ammonia and 75.2% total nitrogen removal. By separating microbial communities within the system, it was able to manage fluctuations in influent quality while keeping the effluent total nitrogen below 6.2 mg L^−1^ [43]. A dual-stage packed bed bioreactor operated efficiently with moderate dissolved oxygen levels (3.5–4 mg L^−1^) and a 2-day hydraulic retention time. It removed 94% of ammonium and 99% of phenol by using sequential oxic and anoxic zones to support both denitrification and anammox processes [39].

PD/A systems link nitrite production from denitrification with anammox, achieving 93–98% total nitrogen removal. These systems work across a wide temperature range, functioning (10–40 °C) and avoiding the aeration energy demands of conventional systems [44]. The structured-bed hybrid baffled reactor (SBHBR) with polyurethane foam beds treated dairy wastewater (COD/TN = 0.2 ± 0.01), removing 98.1% of COD and 80.9% of nitrogen. It maintained stable performance through intermittent aeration and regular sludge removal [37].

Hybrid systems have also been used for the co-removal of nitrogen and organic pollutants. An MBR-Anammox system removed more than 90% of BPA across concentrations ranging from 0.001 to 10^4^ μg L^−1^), while keeping nitrogen removal unaffected. At low BPA levels, EPS production and enzyme activity (HAO, AMO) increased, whereas high BPA levels led to microbial adaptation (e.g., *Candidatus Kuenenia*) and gene upregulation (hzsB, nirS) [26]. An electrolysis-integrated sequencing batch biofilm reactor (E-SBBR) achieved >94.5% NH_4_^+^-N and >90.8% TN removal from low C/N kitchen wastewater using electro-anaerobic hydrogen generation to support autotrophic denitrification and suppress NOB, eliminating external carbon requirements [45].

To better understand the diversity and applicability of various Anammox-based treatment configurations, Table 1 presents a comparative summary of key systems discussed so far. This includes their operational features, strengths, limitations, and suitable wastewater types.

### 4.2. Membrane Fouling Control Strategies

**Figure 3:**
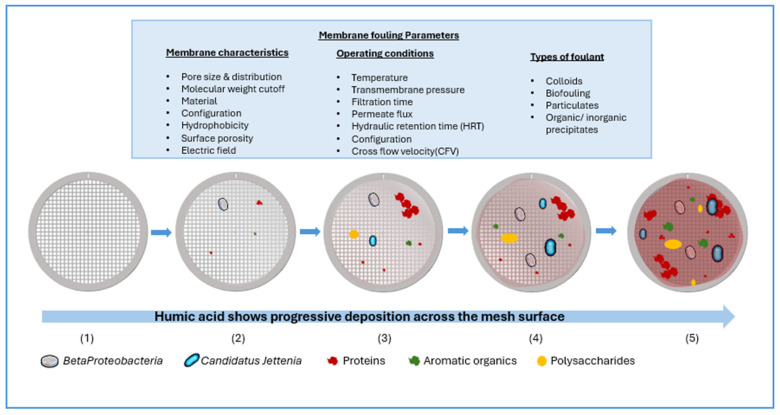
Stages of membrane fouling and the parameters affecting membrane fouling. Redrawn, inspired by [49].

Membrane fouling in anammox-MBR systems is primarily caused by EPS-driven sludge aggregation and biofilm formation. It progresses in three stages: initial (0 < Membrane fouling rate (MFR) < 24 × 10^12^ m^−1^), transition (24–34 × 10^12^ m^−1^), and maturity (34–105 × 10^12^ m^−1^). In early fouling, humic acid in soluble microbial products (SMP-HA), associated with *Betaproteobacteria* and Caldilineae (22 genes), predominates. Later stages are dominated by extracellular proteins (EPS-PN) linked to *Candidatus Jettenia* and the *thrC* gene. Quorum sensing and quenching mechanisms further influence these stages [49]. Anammox sludge contains tightly bound EPS with strong hydrophobicity and a high protein-to-polysaccharide (PN/PS) ratio, both promoting biofilm formation. Therefore, characterizing and managing EPS is vital for stable performance [50]. Figure 3 shows the stages of fouling and parameters affecting membrane fouling.

Membrane material selection significantly affects fouling. Nylon mesh membranes showed better fouling resistance, longer cycles, and stable nitrogen removal than polyvinylidene fluoride (PVDF), especially under low hydraulic retention time (HRTs). Biochar addition in anaerobic systems improved sludge granulation and reduced transmembrane pressure by 30–50%, while boosting methane production by 15–30%. This dual benefit enhances AnMBR performance at COD > 4000 mg L^−1^ [46].

Surface modifications have also been employed. Hydrophilic polyvinyl alcohol coatings on nylon improved flux and reduced foulant accumulation, though excessive coating increased resistance due to structural changes in the fouling layer [51]. CdS/g-C_3_N_4_/rGO-modified membranes exhibited superior anti-biofouling by enhancing hydrophilicity, degrading organics, and inactivating biofilm bacteria via visible-light photocatalysis, thereby reducing transmembrane pressure rise [14].

Microbiological immobilization using carriers like magnetic porous carbon microspheres reduced hydrophobic metabolite deposition, transmembrane pressure, and various types of fouling, while enhancing bacterial retention [28]. Carbon-based biocarriers (e.g., activated carbon, biochar) further aid by adsorbing EPS, scouring membrane surfaces, and promoting stable biofilm formation [29].

Chemical cleaning using NaClO-based chemically enhanced backwashing (CEB) effectively removed polysaccharide-rich foulants. Concentrations of 1495-96 mg L^−1^, particularly around 298 mg L^−1^, were optimal. As polysaccharides accumulate more easily than proteins, precise control is crucial to avoid harming microbial communities [52].

Mechanical innovations also help. Rotating flat-sheet MBRs (RFMBRs) increased shear, reduced biofilm, and promoted granules with lower EPS, sustaining nitrogen removal [53,54]. Similarly, umbrella-shaped modules allowed 105 days of stable operation with minimal cleaning by limiting biofilm buildup and keeping bacteria in suspension [55]. The rotating biological contactor-MBR (RBC-MBR) reduced excess sludge by 45% while maintaining membrane stability. Rotating mesh carriers supported metazoans that degrade fouling agents, with only an 8% increase in energy use compared to A/O-MBRs [56].

Predictive modeling supports fouling control. A backpropagation (BP) neural network based on advanced Derjaguin-Landau-Verwey-Overbeek (XDLVO)-derived adhesion energy data accurately forecasted fouling potential, offering a proactive tool for operational planning in anammox-MBRs [12].

In summary, all studies agree that EPS and biofilms cause fouling [50,51]. Solutions like membrane coatings [51] and photocatalytic membranes [14] work well. However, other methods have trade-offs. For example, the best chemical cleaning dose for the membrane [52] might be too strong for the delicate anammox bacteria [57]. Also, while adding carriers reduces fouling [28,29], their cost at a large scale is still unclear.

### 4.3. Perspective on Configuration Selection and Fouling Mitigation

Based on comparative analysis, PD/A emerges as the most robust configuration for mainstream applications due to its operational stability across wide temperature ranges and inherent handling of nitrate, effectively circumventing the persistent NOB competition challenges that plague PN/A systems [37,44]. Furthermore, the implementation of anammox for side-stream treatment (e.g., of sludge dewatering reject water) provides a critical plant-wide advantage. By removing nitrogen autotrophically, the process minimizes the recirculation of oxidized nitrogen to the mainstream. This directly reduces the energy demand for aeration and the need for an external carbon source for denitrification in the mainstream process, leading to significant operational cost savings by reducing both aeration energy and the purchase of external carbon sources [10]. For fouling control, an integrated strategy is paramount. The most favorable approach combines suspended carriers for biofilm management and foulant adsorption [28,29] with advanced photocatalytic membranes for in situ foulant degradation [14]. This physical-biological-chemical synergy, guided by predictive fouling models [12], offers a sustainable path to mitigate fouling while protecting the delicate anammox community, thereby facilitating the broader adoption of anammox-MBR technology.

## 5. Effects of Environmental Inhibitors and Conditions

The efficient operation of anammox-MBR systems is highly sensitive to environmental conditions. Deviations from optimal parameters, caused by chemical inhibitors [57,58,59] or shifts in factors like temperature [60] and pH [61], directly compromise nitrogen removal efficiency. Furthermore, these deviations pose significant operational challenges by accelerating membrane fouling and prolonging system recovery, thereby increasing maintenance demands and the risk of process failure.

### 5.1. Chemical Inhibitors

Environmental inhibitors such as 3,4-dimethylpyrazole phosphate (DMPP) significantly affect anammox bacterial activity in wastewater treatment. Even at 5 mg L^−1^, DMPP sharply reduces *Candidatus Kuenenia* populations and nitrogen removal by inhibiting key enzymes involved in ammonium transport and hydrazine synthesis [57]. Other inhibitors include heavy metals like Cu^2+^, Zn^2+^, and Mn^2+^, which show concentration-dependent effects, enhancing activity at low levels but becoming toxic beyond the half-inhibiting concentration (IC_50_) values of ~30 mg L^−1^ (Cu), ~25 mg L^−1^ (Zn), and ~4.83 mg L^−1^ (Mn) [58]. Aromatic compounds such as quinoline, common in industrial wastewater, also impair anammox activity at low concentrations (IC_50_ ~13.07–31 ± 6 mg L^−1^), damaging membrane integrity and reducing metabolic performance [59].

### 5.2. Operational Conditions

Operational conditions such as pH and temperature also play critical roles. Production of characteristic ladderane lipids by anammox bacteria, which protect cellular structures, increases at a slightly alkaline pH (~7.5). While this enhances cell integrity, higher pH levels can stress bacterial cells, necessitating gradual acclimatization to maintain stable performance. Maintaining optimal pH supports ladderane synthesis and improves nitrogen removal efficiency [61]. Temperature also plays a pivotal role, with peak activity near 35 °C and minimal N_2_O emissions [60]. The suppression of N_2_O is a key sustainability advantage, as N_2_O is a potent greenhouse gas with a global warming potential approximately 300 times that of CO_2_, as highlighted in the abstract [62]. This range suits warm climates across the equatorial zone, reducing the need for external heating. However, temperatures exceeding 40 °C risk enzyme denaturation and performance decline. Any deviation from optimal temperature, whether lower or higher, decreases nitrogen removal efficiency and may fully inactivate the bacterial population if unregulated [60]. Temperatures in warm climate regions are more advantageous for implementing mainstream PN/A [63].

### 5.3. Nitrite and Organic Carbon Impacts

High nitrite concentrations (>243 mg N L^−1^) disrupt *Candidatus Brocadia sapporoensis* by impairing DNA synthesis and cell wall formation, though basic activity may continue. Recovery often relies on amino acid and cofactor exchange between microbial partners, highlighting the importance of cross-feeding and energy allocation [64].

Organic carbon affects nitrogen removal differently across reactor types. In biofilm-based systems, it may suppress anammox activity but provides a protective matrix that offers resilience. In contrast, activated sludge systems benefit from organic carbon through the stimulation of heterotrophic denitrifiers. Interestingly, at COD/NH_4_-N ratios of 1.6–3.3, phenol-degrading heterotrophs coexist with anammox bacteria, enabling simultaneous removal of phenol (up to 900 mg L^−1^) and nitrogen. Propionate, commonly found in pretreated wastewater, further enhances nitrogen removal via mixotrophic *Ca. Brocadia sapporoensis* through DNRA, highlighting strain-specific metabolic flexibility [31,39].

### 5.4. Antibiotic Stress and Adaptation

Antibiotics such as ciprofloxacin and sulfamethoxazole significantly disrupt EPS and quorum sensing, impairing anammox metabolism. In solar-powered MBRs, although 87–94% of these antibiotics were removed, NH_4_^+^-N removal dropped by 15–20%, and protein-rich EPS production increased (L-EPS/T-EPS ratio 2–3 times). Surprisingly, anammox and AOB populations rebounded, with 25 genera detected, indicating adaptation under long solids retention times (>80 days) [65,66]. Recovery mechanisms include bioaugmentation with resistant strains, EPS-mediated antibiotic adsorption, acquisition of resistance genes, and operational optimization (e.g., MBR integration).

### 5.5. Microbial Resilience and Recovery

Anammox bacteria exhibit slow growth, with doubling times ranging from 11 to 30 days, posing challenges during reactor startup or recovery after inhibition [67]. Fluctuations in solids retention time (SRT) also impact stability; for example, reducing SRT from 50 to 28.5 days caused a decline in *Brocadiales* abundance from 39.8% to 1.4%, while copiotrophic organisms (e.g., *Rhodospirillales*, *Sphingobacteriales*) proliferated. Functional redundancy allowed microbial recovery within 52 days through exopolysaccharide biosynthesis and quorum-sensing reactivation [68]. Biofilm-based strategies, such as using polyurethane carriers, enabled stable biofilm formation within 73 days, achieving >97% NH_4_^+^ and NO_2_^−^ removal [69]. EPS addition and iron-based nanomaterials have also been shown to accelerate startup [70].

In summary, anammox bacteria in biofilms are much tougher against inhibitors like BPA [26] and antibiotics [65] than free-floating cells, which are easily harmed by chemicals like DMPP [57]. Also, while toxins cause initial problems, the bacteria can often adapt and recover over time [65,68]. The effect of organic carbon is mixed; it can be either food or poison, depending on the specific bacteria and system setup [31,39].

## 6. Applications in Challenging Wastewaters

Anammox-MBR systems show significant promise for treating challenging wastewater, particularly saline and high-strength organic streams. Systems utilizing marine anammox bacteria like *Candidatus Scalindua* sp. AMX11 has demonstrated effective treatment [1], achieving over 90% nitrogen removal and stable long-term operation. This offers a sustainable solution for unconventional streams such as seawater toilet flushing effluents. Under low-nitrogen conditions, anammox-MBRs enhance microbial metabolism and interactions, boosting biomass yield and nitrogen removal efficiency to overcome biomass limitations [30]. Furthermore, ^15^N isotope tracing revealed metabolic flexibility in *Candidatus Scalindua* sp. AMX11, showing higher NO_2_^−^ removal (averaging 1.61 mol per mole NH_4_^+^ removed) and lower NO_3_^−^ production (averaging 0.04 mol per mole NH_4_^+^ removed) via respiratory ammonification. This enables NO_2_^−^ reuse, explaining the bacteria’s resilience at 1.2% salinity and 2 mM acetate concentrations [1].

Anammox technology has also been successfully applied to high-strength leachates, which pose treatment challenges due to elevated organic and nitrogen loads. A lab-scale upflow anaerobic sludge blanket (UASB)-MBR sequence removed over 90% of COD and total Kjeldahl nitrogen (TKN). Subsequent treatment with SHARON and anammox reactors achieved effective nitrogen removal, demonstrating that this combined process is viable for high-strength leachate management [71]. Full-scale applications confirm the viability of anammox integration in landfill leachate treatment. A renovated anaerobic + pre-aeration + anammox + MBR process (150 m^3^ d^−1^ capacity) achieved 94.07 ± 1.26% nitrogen removal without carbon addition, with anammox contributing 26.46 ± 1.10% of total removal. Anammox bacteria abundance reached 2.17% (floc sludge) and 2.57% (biofilm), while the simultaneous partial nitrification and denitrification (SPND)-anammox combination reduced treatment costs by 18.51% compared to conventional systems. This confirms the economic and technical feasibility of retrofitting existing plants with anammox-MBR systems [10].

Anammox-MBRs effectively treat high-strength wastewater containing both nitrogen and organic pollutants. For example, systems handling landfill leachate with BPA achieved simultaneous nitrogen removal (Total nitrogen removal rate (TNRR): 0.273–0.290 kg N m^−3^ d^−1^) and >90% BPA degradation, driven by microbial synergy and EPS-mediated protection [26]. Kitchen wastewater treatment benefits from hybrid anammox systems, where multi-stage anoxic/aerobic—anaerobic/aerobic/anoxic (A/O-AOA) configurations coupled with anammox achieve >90% N/P removal despite high COD (3000–5000 mg L^−1^) and oil content. Staged carbon utilization is key: initial aerobic phases degrade organics, while subsequent anammox targets residual nitrogen, enabling effluent reuse standards [72].

The anammox process in sludge dewatering filtrate treatment achieved a secondary 20% microplastic (MP) reduction (from 90.01 ± 1.11 to 72.23 ± 0.82 items L^−1^), following an initial 31% removal by chemical phosphorus removal (via PAM). The primary removal mechanisms are the transfer and entrapment of MPs within the dense anammox granules and biofilms, as well as adsorption onto the extracellular polymeric substances (EPS) matrix. The long sludge retention time may also facilitate potential slow biodegradation. This highlights anammox’s ancillary role in co-managing nitrogen and MPs in wastewater side streams [73,74].

To address concentrated municipal wastewater treatment challenges, a membrane-integrated process combining anaerobic digestion and nitritation-anammox (CANON) enables energy-neutral operation at ambient temperature. This system achieved ~96% COD removal with concurrent methane production and 81% total nitrogen removal. Rapid startup, efficient biomass retention, and low net energy consumption (0.09 kWh m^−3^) make it a promising, cost-effective, and sustainable solution [13].

Pilot-scale demonstrations support integrating anammox into mainstream municipal treatment. A combined high-rate activated sludge, reverse osmosis (RO), and partial nitritation-anammox (PN/A) system recovered 75% of municipal wastewater and subsequently treated, achieving 90% total nitrogen removal. Operating without chemical additives, it reduced energy use and proved economically suitable for water-scarce regions [36]. A 38 m^3^ pilot-scale sludge fermentation system achieved rapid in situ anammox enrichment (3460-fold AnAOB increase in 185 days) via H_2_ production from waste-activated sludge. This boosted nitrogen metabolism electron pools by 200%, enabling 61.9% NH_4_^+^ removal alongside 72.1% sludge reduction, demonstrating scalable resource recovery [75]. Similarly, anammox membrane bioreactors (Anammox-MBRs) for mainstream sewage achieved stable COD and nitrogen removal. Despite temperature fluctuations, the system maintained >70% total nitrogen removal while reducing aeration demand, showing operational resilience and cost-saving potential [76].

However, anammox application in certain complex wastewaters presents challenges. Treating thermal hydrolysis pre-treated side streams (THPS), characterized by high organic content, led to DNRA bacteria proliferation, inhibiting anammox activity. While some cooperative nitrogen removal via DNRA–anammox interactions occurred, excessive organic loads caused anammox biomass washout, highlighting the need for careful process design and organic load control in side streams [77]. In petrochemical wastewater treatment, PN/A systems achieved stable nitrogen removal for diluted acrylic fiber wastewater at a 30% volume ratio. Selective inhibition of AOB in flocs was observed, while anammox bacteria in granules remained initially active. However, long-term granule disintegration eventually reduced performance, emphasizing the importance of maintaining sludge morphology and wastewater dilution for PN/A stability [78].

For printing and dyeing wastewater (PDW), Fenton pretreatment coupled with A/O-MBR demonstrated superior removal of persistent pollutants-achieving 90% COD and 79% AOX (halogenated organics) removal versus 67% and 35% without pretreatment. Oxidative pretreatment reduced membrane fouling while maintaining reasonable costs (1.26–1.38- USD/t), offering a viable solution [79]. An anaerobic membrane bioreactor (AnMBR) coupling sulfammox (sulfate-dependent ammonium oxidation) and sulfide-driven autotrophic denitrification (SDAD) achieved simultaneous sulfate (45%) and nitrogen (31%) removal from rubber wastewater over 225 days. Key microbes (*Desulfovibrio*, *Sulfurospirillum*) enabled this low-energy, chemical-free treatment [48]. A pilot-scale AnMBR treating real seafood wastewater (500 L d^−1^) achieved 61.04% COD removal and met discharge standards despite challenging nitrogen levels. It maintained stable operation (Transmembrane pressure (TMP): 0.66 bar; flux: 18.2 L m^−2^ h^−1^) with robust sludge characteristics (MLSS: 11.5 g L^−1^, SVI: 20) over two months of operation [47]. Table 2 shows the recent advances in anammox-based MBR and its configurations and operational highlights.

## 7. Challenges and Future Perspectives

Anammox-MBR technology still has limits for widespread application, despite its achievements. Membrane fouling is still a significant problem, worsened by hydrophobic metabolites from anammox bacteria. This necessitates advanced surface treatments and phase-specific control strategies, such as targeting SMP-HA producers (*Betaproteobacteria*) early and EPS-PN synthesis via *thrC* or *Candidatus Jettenia* at maturity [49,86,87,88]. Stability is also restricted by temperature sensitivity, with activity decreasing markedly below 10 °C, and by the persistent challenge of suppressing nitrite-oxidizing bacteria(NOB) in mainstream applications, where dynamic conditions often overwhelm conventional control strategies [89]. Furthermore, while systems can withstand low levels of inhibitors like BPA (10 μg L^−1^), higher concentrations limit AMO and NOB activity [26].

Promising pathways to address these challenges are emerging. Hybrid MBRs demonstrate a strong ability to handle load changes and achieve simultaneous nitrogen and phosphorus removal [40]. Novel nitrogen removal pathways like Feammox, Sulfammox, and Mnammox offer alternatives but require further validation [90]. Similarly, Microbial electrochemical technologies (METs) and quorum-sensing-based strategies present opportunities for nitrogen recovery and enhanced process stability, though they are currently limited by cost and scalability [33,91]. The metabolic versatility of mixotrophic anammox strains shows great promise for treating low C/N wastewater [31].

Therefore, Future research must be strategically directed. The priority is to develop advanced anti-fouling membranes, cultivate cold-resistant strains, and implement AI-based process optimization. It is also important to conduct comprehensive life-cycle assessments and techno-economic analyses to validate the long-term benefits and guide full-scale implementation [88]. Finally, combining anaerobic co-digestion with anammox and PN/A can make wastewater treatment carbon-neutral, potentially cutting emissions by 60–80% and recovering energy between 0.5 and 1.2 kWh m^−3^ [11]. By focusing on these targeted areas, anammox-MBR technology can overcome current barriers and solidify its role as a cornerstone of sustainable wastewater management.

## 8. Conclusions

By fusing the superior biomass retention and operational control of membrane bioreactors with the energy-efficient anammox process, Anammox-MBR technology represents a significant advancement in sustainable nitrogen removal from wastewater. Recent breakthroughs in process configurations, microbial ecology, and fouling control enable faster start-ups, nitrogen removal efficiencies of 70–90%, and enhanced stability, even treating challenging wastewater like saline streams. Innovations such as hybrid reactor designs (e.g., SNAD, PD/A, MABR), membrane surface modifications (e.g., hydrophilic coatings, photocatalytic membranes), and microaerobic cultivation further improve performance and reduce fouling.

Despite these improvements, key challenges remain for large-scale use. Membrane fouling, worsened by hydrophobic metabolites from anammox bacteria, and maintaining a stable microbial consortium (particularly suppressing NOB) are major hurdles. The sensitivity of anammox bacteria to low temperatures (≤10 °C) and competition from NOB also complicates mainstream applications. Future research must focus on developing cold-tolerant anammox strains, creating advanced anti-fouling membranes, and optimizing microbial management strategies. Successfully addressing these challenges will enable the cost-effective, energy-efficient, and environmentally sustainable large-scale implementation of Anammox-MBR technology, with a significantly lower greenhouse gas footprint compared to conventional processes.

## Figures and Tables

**Table 1:** Comparative summary of anammox configurations and key operational strategies.

Configuration	Key Features	Advantages	Limitations	Suitable Wastewater	References
PN/A	low DO (0.5–1.0 mg L^−1^) nitrite accumulation	low energy no external carbon required	NOB competition sensitive to temperature	side streams municipal	[32,35,41]
PD/A	NO_2_^−^ production via denitrification	works well in low C/N handles nitrate	COD level sensitivity	industrial high-ammonia wastewater	[44]
SNAD	oxygen-limited mixed microbial process	COD and TN removal low aeration	requires microbial balance	organic-rich domestic wastewater	[26]
CANON	single reactor, granular sludge	efficient side-stream N removal	sensitive to temperature; requires granules	sludge liquor digester effluent	[13]
MABR	biofilm on oxygen-permeable membranes	low N_2_O emissions efficient biofilm control	high cost DO tuning complex	low DO influents acidic/cold streams	[6]
AnMBR	anaerobic with membranes biogas production	low sludge energy recovery	fouling slower startup	high-strength industrial or kitchen wastewater	[46,47,48]

**Table 2:** Recent advances in anammox-based MBR: configurations, operational highlights & performance.

Type of Water Stream	Configuration	Scale	TN or NRR/COD Removal	Key Operational Highlights	References
High-strength leachate	UASB-MBR + SHARON-Anammox	Lab-scale	>90% COD and TKN removal	combined anaerobic and aerobic processes effective for high organic & nitrogen loads	[71]
Marine saline, low C/N (*Scalindua* sp.)	Anammox MBR suspension culture	Lab-scale	>90% TN removal, NRR = 0.3 kg N m^−3^ d^−1^ under ~1.2% salinity and 2 mM acetate	high salinity resilience *Scalindua AMX11* enrichment low sludge yield controlled substrate affinity via MBR	[1,30]
Dual-stage packed bed (phenolic industrial)	Sequential oxic–anoxic packed-bed bioreactor	Pilot	~94% NH_4_^+^, 99% phenol removal	handles toxic phenolics synergistic zones 2-day HRT moderate DO (3.5–4 mg L^−1^ )	[39]
MBR Anammox (BPA leachate)	MBR with Anammox for microconstituents	Lab-scale	TNRR ~0.27–0.29 kg N m^−3^ d^−1^; >90% BPA degradation	EPS-mediated protection; gene shift at high BPA emergent *Candidatus Kuenenia*	[26]
Electro-SBBR (kitchen wastewater)	Electrolysis-integrated SBBR	Lab-scale	NH_4_^+^-N > 94.5% and TN > 90.8%	in situ alkalinity & H_2_ for autotrophs no external carbon NOB suppression	[45]
SBHBR (dairy wastewater)	Structured-bed baffled reactor with foam	Pilot	COD 98.1%, TN 80.9% at COD/TN ~0.2	intermittent aeration; sludge scheduling low-cost without external C	[37]
Waters with a low NO_2_^−^/NH_4_^+^ ratio	Standard Anammox MBR enrichment	Lab-scale	Moderate TN removal	*Candidatus Jettenia* enriched (0.01% to 26.19%) low TMP (<4 kPa) SMP—polysaccharides dominate fouling large particle foulants (~200 µm)	[80]
Anammox MABR (acidic pH)	MABR at pH 5.0–5.2	Lab pilot	~2.4 kg N m^−3^ d^−1^ NH_4_^+^ removal	acid-tolerant *Candidatus Nitrosoglobus* suppresses NOB > 200 days N_2_O/NO emissions < 1%	[8,9]
municipal/domestic wastewater	Heated aeration MABR	Pilot	Total: ~70.9%; AOB: 42.8%, Anammox: 28.1%	DO control was challenging due to high membrane air permeability future work should explore lower-permeability materials nitrogen removal aligned with bacterial group abundance.	[81]
Municipal wastewater	Hybrid MABR–AnMBR	Review (lab-scale cases)	Energy-neutral; enhanced TN removal and biogas production	localized O_2_ delivery supports partial nitrification and anammox reduced sludge methane biogas recovery	[82]
Municipal wastewater (cold climate)	Mainstream Anammox MBR	Pilot-scale	70.4 ± 4.5% at 10-7.5 °C	stable nitrogen removal at low temperature cold-tolerant anammox bacteria grew well demonstrated the feasibility of mainstream anammox in cold climates	[83]
Low-strength wastewater	Anammox–Microalgae PBR	Lab-scale	Up to 99.51% TN removal	microalgae-bacteria symbiosis without mechanical aeration highest TN removal achieved ~99.51%	[84]
High-strength wastewater	Granular Anammox UASB	Lab-scale	NRR up to 38 kg N m^−3^ d^−1^ in 44 days	gradual HRT shortening and nitrogen loading rate (NLR) increase enabled rapid granulation and high-rate anammox enriched *Ca. Brocadia* to 57.1%. EPS and heme c secretion increased granule density.	[85]

## Data Availability

The raw data supporting the conclusions of this article will be made available by the authors on request.
